# Emergence and selection of isoniazid and rifampin resistance in tuberculosis granulomas

**DOI:** 10.1371/journal.pone.0196322

**Published:** 2018-05-10

**Authors:** Elsje Pienaar, Jennifer J. Linderman, Denise E. Kirschner

**Affiliations:** 1 Department of Microbiology and Immunology, University of Michigan Medical School, Ann Arbor, Michigan, United States of America; 2 Department of Chemical Engineering, University of Michigan, Ann Arbor, Michigan, United States of America; Fundació Institut d’Investigació en Ciències de la Salut Germans Trias i Pujol, Universitat Autònoma de Barcelona, SPAIN

## Abstract

Drug resistant tuberculosis is increasing world-wide. Resistance against isoniazid (INH), rifampicin (RIF), or both (multi-drug resistant TB, MDR-TB) is of particular concern, since INH and RIF form part of the standard regimen for TB disease. While it is known that suboptimal treatment can lead to resistance, it remains unclear how host immune responses and antibiotic dynamics within granulomas (sites of infection) affect emergence and selection of drug-resistant bacteria. We take a systems pharmacology approach to explore resistance dynamics within granulomas. We integrate spatio-temporal host immunity, INH and RIF dynamics, and bacterial dynamics (including fitness costs and compensatory mutations) in a computational framework. We simulate resistance emergence in the absence of treatment, as well as resistance selection during INH and/or RIF treatment. There are four main findings. First, in the absence of treatment, the percentage of granulomas containing resistant bacteria mirrors the non-monotonic bacterial dynamics within granulomas. Second, drug-resistant bacteria are less frequently found in non-replicating states in caseum, compared to drug-sensitive bacteria. Third, due to a steeper dose response curve and faster plasma clearance of INH compared to RIF, INH-resistant bacteria have a stronger influence on treatment outcomes than RIF-resistant bacteria. Finally, under combination therapy with INH and RIF, few MDR bacteria are able to significantly affect treatment outcomes. Overall, our approach allows drug-specific prediction of drug resistance emergence and selection in the complex granuloma context. Since our predictions are based on pre-clinical data, our approach can be implemented relatively early in the treatment development process, thereby enabling pro-active rather than reactive responses to emerging drug resistance for new drugs. Furthermore, this quantitative and drug-specific approach can help identify drug-specific properties that influence resistance and use this information to design treatment regimens that minimize resistance selection and expand the useful life-span of new antibiotics.

## Introduction

Tuberculosis (TB) is caused by infection with *Mycobacterium tuberculosis* (Mtb) and remains a global public health challenge. In 2015 there were 10.4 million new TB cases reported worldwide, 480,000 of which were classified as multi-drug resistant (MDR) [1], defined as simultaneously resistant to the first-line antibiotics isoniazid (INH) and rifampin (RIF). Trends indicate that MDR-TB incidence is rising [1], and it is therefore vital to understand the mechanisms of resistance to slow the spread of MDR-TB and to minimize the emergence of resistance to new drugs.

Patients infected with Mtb can be placed on a spectrum between two clinical outcomes: (1) active TB disease with clinical manifestations; and (2) clinically latent infection where patients show no signs of disease, but still harbor bacteria within granulomas [2]. The vast majority of patients (~90%) will develop latent infection, and patients can progress along the spectrum, sometimes developing active TB disease decades after their initial infection. Antibiotic treatment is recommended whether a patient has active or latent infection [3, 4]. Standard treatment of clinically active TB consists of combination therapy with 2 to 4 antibiotics, including INH and RIF, given simultaneously over the course of 6 to 9 months [3]. Standard TB treatment is 83% effective globally [1]. INH monotherapy spanning 9 months is recommended for the treatment of latent (asymptomatic) Mtb infection. Treatment of latent TB reduces the risk of subsequent progression to active TB disease [4], but could be associated with increased risk of resistance [5, 6].

Mtb infection leads to the formation of multiple granulomas in host lungs, lymph nodes and extrapulmonary sites [7, 8]. Granulomas are complex sites of infection in TB, and each is comprised of a dense collection of host immune cells, bacteria and dead cell debris (caseum). Drug penetration into granulomas is key to treatment success. Experimental and computational studies indicate that standard INH and RIF doses result in sub-therapeutic concentrations inside TB granulomas, which contributes to poor treatment outcomes [9–11]. Sub-therapeutic exposure has been linked to selection of drug resistance [12, 13]. Understanding the factors that lead to drug resistance in the context of bacterial, immune, and antibiotic dynamics is critical to improving treatment.

**Fig 1:**
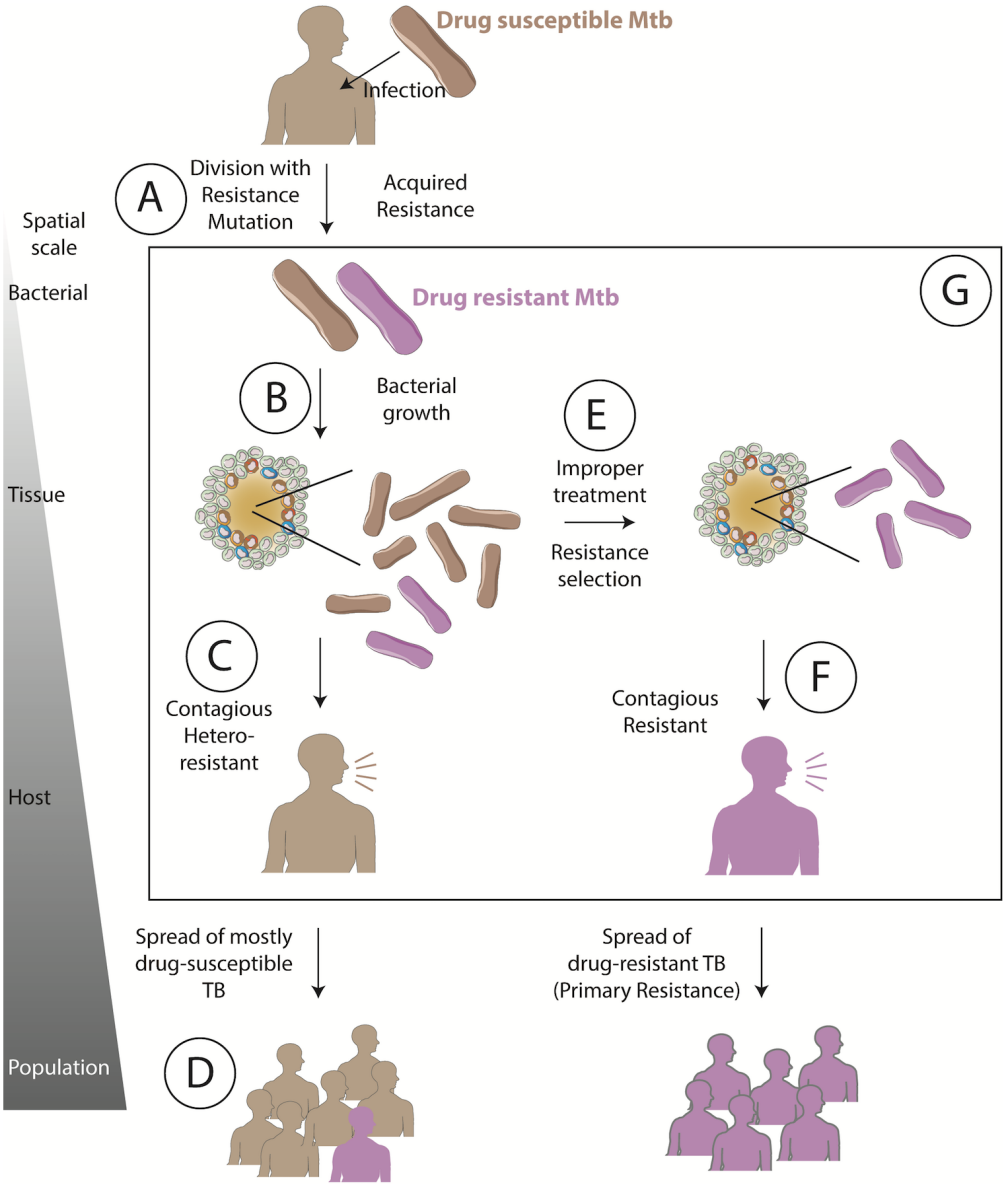
Antibiotic resistance emergence and selection over bacterial through population scales. Resistance develops upon mutation conferring genetic resistance (A). Resistant bacteria grow (B) within the host, giving rise to hetero-resistance (C), which can spread resistant bacteria to others (D). Improper treatment (E) selects for resistant bacteria, resulting in drug-resistant TB (F), which can spread in a community. (G) The influence of bacterial and drug dynamics on resistance emergence and selection in the context of granulomas remains unclear. Arrows between scales represent a series of potential events, but influences can occur in both directions.

We use a computational systems pharmacology approach to simulate Mtb infection, resistance acquisition, and antibiotic treatment so that we can predict INH- and RIF-resistance. While resistance can be defined for individual bacteria—a single bacterium is either susceptible or resistant to an antibiotic—drug-resistant (DR)-TB disease and epidemics emerge over interconnected bacterial, granuloma, host and population scales [14] (Fig 1). The current DR-TB epidemic can be viewed as the result of: genetic resistance occurring at the bacterial scale through mutation when susceptible Mtb divide (Fig 1A) (*acquired drug resistant TB*); these resistant bacteria becoming a significant proportion of the total bacterial population within a single granuloma at the tissue scale (Fig 1B); spread of one or more resistant bacteria to a new host (Fig 1C) (*primary drug resistant TB*); the resistant bacteria establishing infection in a new host; and this process repeating to generate and spread DR-TB at the population scale (Fig 1D). At each of these scales, transition to the next scale depends on multiple factors including the number of resistant bacteria, fitness cost of resistant mutations, host immunity and epidemiological factors.

Epidemiological and computational studies estimate that primary MDR-TB (rather than acquired MDR-TB) is currently responsible for 48–99% of MDR-TB cases in several countries, including the Philippines, Russia and South Africa [15–17]. These results indicate that limiting acquired resistance alone would not eliminate DR-TB, but could potentially change the global trend of increasing incidence of DR-TB, especially in countries where half of DR cases are still due to acquired resistance [15–17]. Furthermore, as new antibiotics are developed and used, it is vital to minimize acquired resistance to these antibiotics in an effort to forestall the dominance of primary resistance. Therefore, in this work we focus on acquired drug resistance.

Antibiotics are understood to apply selective forces on the bacterial population along its path to resistance, providing resistant Mtb an evolutionary advantage by increasing their proportion in the total bacterial population (Fig 1E) [18]. If this advantage is strong enough to overcome fitness costs incurred by a resistance mutation(s), the probability of transmitting resistant Mtb is increased when treatment fails (Fig 1F). However, the role of host immunity and antibiotic dynamics within granulomas remains unclear (Fig 1G) [14, 19].

Other studies have importantly bridged bacterial and population scales; however, as yet none have explicitly considered granuloma dynamics. Experimental and clinical studies have greatly advanced our understanding of resistance at the bacterial and population scales [20–25]. INH resistance most commonly arises through mutations in katG (the activator of INH) or inhA (the target of INH) [26]. RIF resistance occurs through mutations in the resistance determining region of the rpoB gene encoding the β subunit of the bacterial RNA polymerase [27]. INH resistance is found in 1 out of 10^7^ to 10^8^ bacteria, and RIF resistance in 1 out of 10^8^ to 10^10^ bacteria in liquid culture [28–31]. Beijing strains of Mtb have been shown to accumulate mutations more quickly than other strains [32], and Mtb lineage has been found to influence disease manifestation in combination with host genetics [33, 34]. Computational approaches have been used to understand how drug susceptible and drug resistant bacterial populations interact and compete. Such studies have included antibiotic pressure, fitness costs, bacterial evolution, and/or host immunity [14, 32, 35–48].

In this work we focus on the relatively understudied knowledge-gap between bacterial and host scales (Fig 1), using our systems pharmacology approach to ask: 1) assuming infection with a drug-susceptible Mtb strain, when does resistance first emerge within granulomas and with what probability? and 2) how do INH and RIF treatment influence the survival of these resistant bacteria within granulomas? Understanding these early dynamics in the development of resistance from bacterial to population scales will be vital to designing new treatment regimens and extending the useful lifespan of new drugs.

## Methods

We use our spatiotemporal computational model, *GranSim*, to simulate the formation of lung granulomas in response to Mtb infection, as well as treatment with INH and RIF. *GranSim* is continuously curated and calibrated to per-granuloma spatial and temporal data obtained from Mtb-infected non-human primates [7, 8, 49, 50]. For the first time, we implement bacterial mechanisms necessary to study drug resistance within granulomas: mutation to acquire resistance to INH and/or RIF, fitness advantages and costs associated with susceptibility and resistance phenotypes, and mutation to overcome resistance fitness costs. We perform uncertainty and sensitivity analyses on the system to determine factors leading to resistance. Dynamics and resistance emergence may differ between granulomas in different physiological compartments (lung, lymph node and other extrapulmonary sites). Here, we restrict our analysis to lung granulomas.

### Model structure

#### Host immune mechanisms

**Fig 2:**
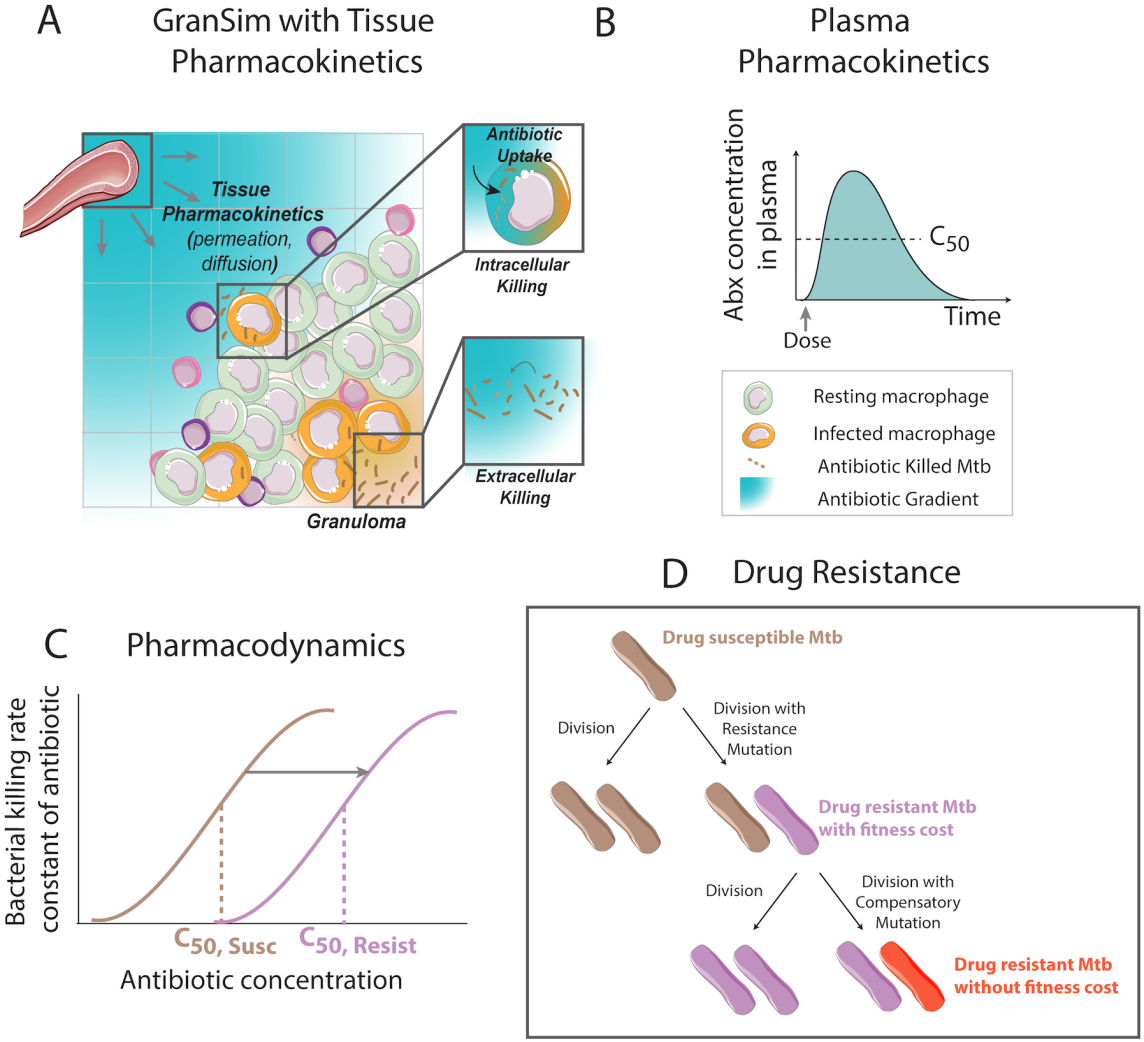
Host immunity, PK and PD model structure in GranSim. (A) Granuloma computational model (GranSim) simulates spatial and temporal host immunity dynamics and granuloma formation within a primate lung. Tissue pharmacokinetics (PK) that lead to an antibiotic distribution within a granuloma are also captured. (B) The plasma PK model predicts plasma dynamics following recommended dosing with INH and RIF. (C) PD is modeled using an Emax curve to calculate the bacterial killing rate constant for each antibiotic as a function of antibiotic concentration. The location of PD curve is determined by the bacterial resistance state, with resistant bacteria having higher effective concentrations (C_50,Resist_) compared to antibiotic-susceptible Mtb (C_50,Susc_). (D) Upon bacterial division, bacteria may mutate to acquire antibiotic resistance, incurring a fitness cost. Resistant bacteria have the opportunity to acquire compensatory mutations to overcome the fitness cost.

*GranSim* couples agent-based, ordinary differential equation and partial differential equation models into a single hybrid, multi-scale computational framework [10, 51–54]. Briefly, the model incorporates host immune cell and bacterial dynamics in a two-dimensional simulation grid representing a portion of lung tissue (Fig 2A). Host immune cells (“agents”) in the model include: T cells (effector, regulatory or cytoxic) and macrophages (resting, infected, chronically infected or activated). T cells and macrophages produce cytokines (TNFα, IFNγ and IL10) and chemokines (CCL2, CCL5, CXCL9) that diffuse on the simulation grid and influence host cell states (e.g. macrophage activation) and chemotaxis. Immune cells interact with each other (e.g. T cells activating macrophages) and with bacteria (e.g. phagocytosis). *GranSim* does not yet include all cells and molecules known to be present in the granuloma e.g. neutrophils and B cells [55, 56]. The roles of some of these are not clear (e.g B cells and humoral immunity), and thus cannot yet be included in sufficient detail. For others, their roles are lumped in with existing cell types (e.g. neutrophils) [57]. The model is continuously curated and easily adapted as more data become available. Nonetheless, extensive calibration to multiple datasets ensures that the model reflects *in vivo* infection dynamics.

#### Bacterial growth and death

Bacterial growth and death are implemented as in [58], but with bacterial growth-rate coarse-grained to reduce computational cost [59]. Briefly, as in [58], bacteria are simulated as individual agents within *GranSim* that grow, divide, die or are phagocytosed by host macrophages. Each bacterial agent tracks its size as it grows, and when this size reaches a threshold it divides into two new bacteria. In [58], bacterial growth rate constants were determined by a constraint-based model of Mtb metabolism driven by dynamic infection-driven nutrient levels at specific locations within granulomas. Here, the coarse-grained approach is taken in which bacterial growth rate constants are model parameters, specific to the location and metabolic state of the bacteria [59]. Bacteria in the model are divided into three groups: intracellular replicating, extracellular replicating, or extracellular non-replicating. Extracellular non-replicating bacteria are located in caseum and reflect metabolic adaptation of the bacteria to low-oxygen conditions in the caseum [58, 60, 61]. These bacteria remain viable but in a non-replicating state. While this approach sacrifices detail in nutrient-driven bacterial growth, it retains variable growth rate constants for different bacterial locations. The combination of host immunity and bacterial growth produces *in silico* granulomas within *GranSim*. Model outcomes are calibrated to granuloma data from non-human primates, including bacterial load per granuloma, granuloma size and cytokine knock-out phenotypes [7, 8, 49, 50]. For a full set of model rules and implementation, as well as a model executable and representative parameter files, see malthus.micro.med.umich.edu/GranSim/.

#### Antibiotic treatment

*GranSim* incorporates antibiotic therapy into the framework of granuloma formation and function by simulating plasma pharmacokinetics (PK), tissue PK and pharmacodynamics (PD) (Fig 2A, 2B and 2C) [10, 11]. Plasma PK describe antibiotic concentration changes in plasma over time following multiple doses, and is represented by a system of ordinary differential equations describing a two-compartment model with two transit compartments. Parameters in the plasma PK model describe antibiotic absorption into blood, distribution into peripheral tissues, and elimination through metabolism. Tissue PK describe antibiotic concentration changes in space and time within lung tissue and granulomas, and use ordinary and partial differential equations to capture permeation through blood vessel walls, diffusion in lung tissue, uptake into host cells and degradation.

PD is implemented as a dose-response curve, using an Emax model to translate antibiotic concentration to a killing rate constant (*k*_*kill*_), with C_50_ defining the concentration where the antibiotic reaches 50% of its maximum efficacy, H defining the hill constant (steepness of the dose-response curve), and C denoting the local concentration of antibiotic [62].

$$k_{kill}=E_{max}\frac{C^{H}}{C^{H}+C_{50}^{H}}$$

Antibiotic exposure (a PK metric) is often quantified by calculating the area under the concentration curve (AUC) over a defined time period, usually 24 hours following a dose. Analogous to the concentration curve, one can use the *E*_*max*_ model to construct an ‘effect-curve’ showing the concentration-dependent antibiotic effect over a defined time period. Calculating the area under this effect curve provides a PD metric (area under the effect-curve, AUC_E_) that is a metric of cumulative antibacterial activity over that time period [63]. PK and PD for INH and RIF are calibrated to data from Mtb-infected non-human primates wherever possible. Lesion PK data from Mtb-infected rabbits was used to calibrate lesion PK parameters since non-human primate data was not available. However, rabbit lesion PK data was only used in conjunction with rabbit plasma PK data. Detailed descriptions of the calibration process can be found in [10, 11].

#### Antibiotic resistance

In this work, we implement a new mechanism simulating the emergence and selection of INH-, RIF- and multi-drug (INH and RIF) resistance in a granuloma scale model (Fig 2D). Including genetic resistance in the context of our immune infection model requires bacterial agents to mutate to acquire resistance. We also consider the fitness costs and higher C_50_ values associated with resistance.

We represent genotypic antibiotic resistance by giving each bacterial agent the ability to acquire resistance-conferring mutations upon division. The probability of mutation during each division is equal to: (mutation frequency per base pair per generation) x (number of mutations associated with resistance). Mutations in Mtb accumulate at a similar frequency per base pair per day (~3x10^-10^ mutations/base pair/day), regardless of bacterial generation time, which implies that the per-generation mutation frequency increases with increasing generation time (time between divisions for each bacterium) [64]. We implement this dependence on generation time as follows:

$$p_{m}=1-{\left(1-f_{m}\right)}^{t}$$ (1)

where *p*_*m*_ is the mutation frequency per base pair per generation, *f*_*m*_ is the mutation frequency per base pair per 10-minute computational model time-step (based on [64]), and *t* is the generation time (in number of 10-minute model time-steps) specific to each bacterium.

In this work we consider resistance acquisition through random genetic mutation only. While some antibiotics have been shown to induce resistance mutations in Mycobacteria [65], to our knowledge, this has not been shown for INH or RIF. Phenotypic tolerance induction has been described through various mechanisms including bacterial transcriptional changes in response to host immune responses [66], epigenetics [67, 68], inherent bacterial variability [69, 70], lineage differences [32, 71] and efflux pump induction [72]. These mechanisms are outside the scope of the current work. Our simulations are able to predict the selection of resistant bacteria under chronic suboptimal exposure observed *in vitro*, and specifically in the context of host immunity within granulomas.

Antibiotic resistance is typically associated with a fitness cost [34, 73–76]. We implement this fitness cost as a replicative fitness cost. Other costs associated with virulence or transmissibility apply to the population scale and are outside the scope of this work.[77, 78]. The growth-rate constant for each bacterial agent is calculated as a ‘wild-type’ growth rate (determined by model calibration to non-human primate data [7, 8, 49, 50]) minus the combined fitness costs of resistance as determined by *in vitro* experiments with clinical Mtb strains [76]. Bacterial agents also have the ability to acquire mutations to compensate for fitness costs associated with drug resistance [22, 24, 40, 79–82], which sets the fitness cost to zero for resistance to that drug. We assume (as have others) that acquiring antibiotic resistance does not affect susceptibility of a bacterium to host immune mechanisms, e.g. killing by activated macrophages [83].

### Simulation protocols

We initiate granuloma simulations by placing a single infected macrophage containing one antibiotic-susceptible Mtb bacterium in the center of the grid, and randomly distributing resting macrophages and vascular sources throughout the rest of the grid. We allow the bacterial population to grow, and host immune mechanisms to respond according to *GranSim* rules (malthus.micro.med.umich.edu/GranSim/), resulting in granuloma formation. See Table A in S1 File for baseline host immunity and bacterial growth parameters, and Table B in S1 File for INH and RIF PK and PD parameters. Simulations were performed on Extreme Science and Engineering Discovery Environment (XSEDE), which has peak performance up to 7 Petaflops/sec using XEON PHI co-processors. Simulations in the absence of treatment have runtimes of ~3 hours per granuloma to simulate 200 days of infection. Simulations including antibiotic treatment have runtimes of ~9 hours per granuloma for 180 days of treatment.

Computational studies based on experimentally measured mutation rates for Mtb have suggested that the classical view [35] of pre-existing DR strains subsequently being selected for by drugs likely holds for TB also [32, 46, 47]. Colijn et al expanded the classical Luria Delbruck equations to include bacterial death and fitness costs, concluding that mono-resistance is present with near certainty prior to treatment, and MDR-TB is present with a probability of 10^−5^ to 10^−4^ in previously untreated hosts [46]. Estimates from clinical studies indicate that between 0.1 to 15% of previously untreated patients had INH resistant Mtb [84–88]. Though some of these cases are likely due to primary DR-TB rather than acquired DR-TB, it is conceivable that the earlier studies (before widespread implementation of INH, and spread of INH-R TB), would represent mainly acquired resistance. Taken together, these data indicate that resistance emerges most commonly prior to treatment, and that resistance selection occurs during treatment.

**Fig 3:**
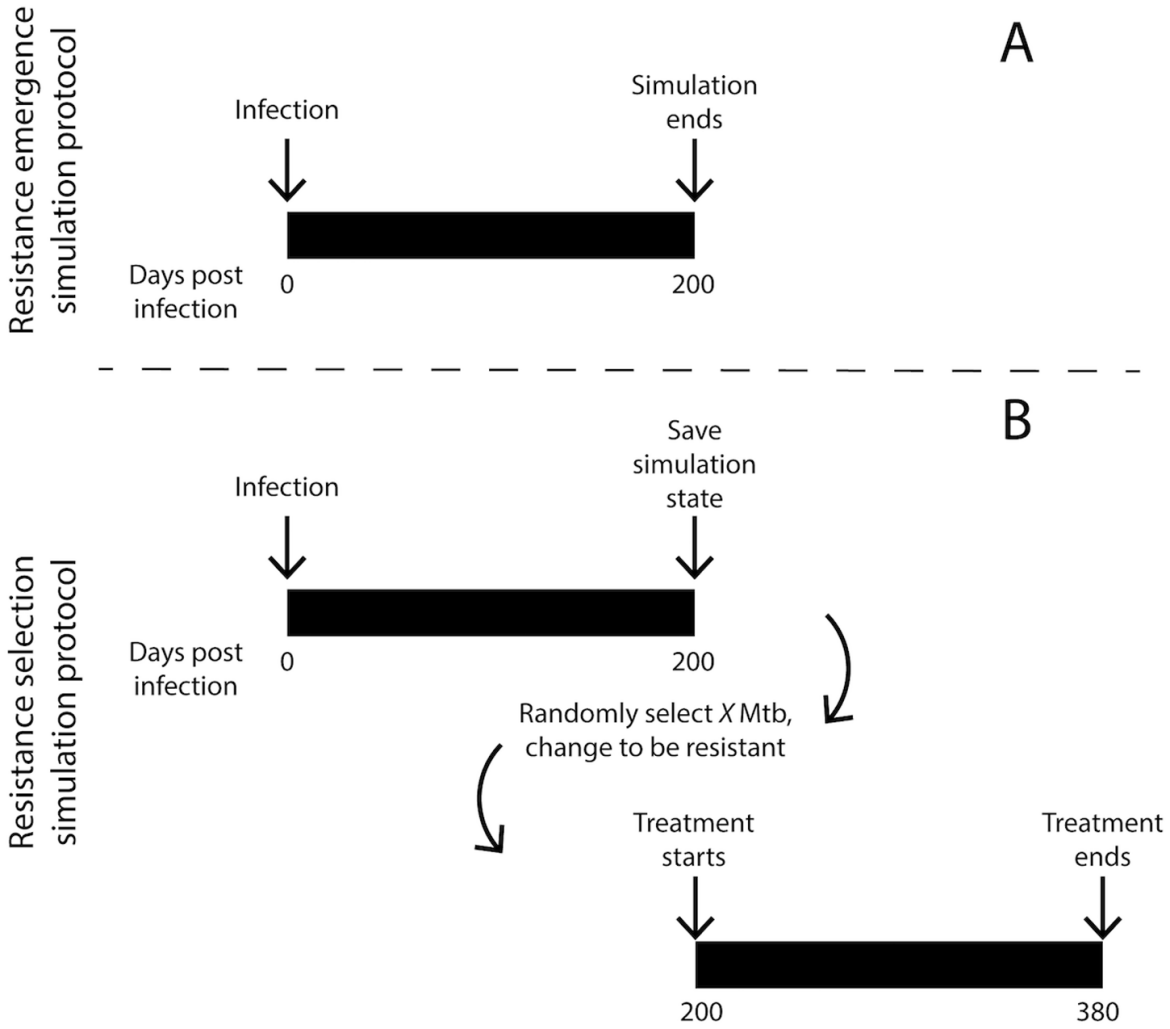
Simulation protocols for resistance emergence (A) and resistance selection (B). (A) Resistance emergence is tracked over 200 days of infection. (B) Resistance selection is simulated by stopping simulations at 200 days post infection, randomly selecting bacteria and changing them to be resistant (X: number of bacteria to be changed to resistant), and then resuming simulations in the presence of antibiotics.

We therefore run two types of simulations: 1) *resistance emergence* simulations in the absence of treatment, tracking mutation and growth of resistant bacteria in the context of host immunity within granulomas, and 2) *resistance selection* simulations tracking survival of resistant bacteria throughout treatment (Fig 3). For both of these simulation types we initiate 500 granuloma simulations. Due to the stochastic nature of *GranSim*, some of these granulomas will naturally sterilize the infection, in agreement with *in vivo* observations [7]. We therefore only include non-sterilized granulomas in our analyses and figures.

#### Resistance emergence simulations

Resistance emergence is a relatively rare event [31], and simulating rare events in *GranSim* is challenging due to the large number of computationally intensive simulations required. For example, if the expected granuloma resistance frequency (Box 1) is 1x10^-5^, then 100,000 simulated granulomas would be required to obtain one granuloma with resistant bacteria. We therefore study the emergence of resistance in the context of spatial and temporal dynamics within *GranSim* using a higher mutation frequency (3x10^-6^ mutations per base pair per day) than that measured in non-human primates (3x10^-10^ mutations per base pair per day) [64]). This approach is akin to experimental studies, e.g. increasing the cognate frequency of T cells to perform two-photon microscopy allows for closer inspection of rare antigen detection events [89].

**Box 1. Resistance frequencies defined by scale.**

In describing resistance, we define the following terms:

1. host resistance frequency (fraction of hosts that have at least 1 resistant bacterium in any of their granulomas)
2. granuloma resistance frequency (fraction of granulomas that have at least 1 resistant bacterium)
3. bacterial resistance frequency (fraction of a bacterial population per granuloma that is resistant)
4. mutation frequency (per base pair frequency of mutation)

For resistance emergence simulations (Fig 3A) we initiate infections at day 0 and observe the formation of granulomas. For every bacterial division, resistance mutations occur with probabilities as described above. For each granuloma, we track model outcomes relevant to resistance emergence (Table 1) over 200 days of infection. Thus, our simulations predict how resistance emerges in granulomas over time, and how the host immune response influences these dynamics.

**Table 1:** Simulation outputs evaluated.

	Resistance emergence simulations	Resistance selection simulations
Model outputs	Numbers of resistant bacteria per granuloma Proportion of non-sterile granulomas with at least one resistant bacterium Location (intracellular, extracellular, caseum) of resistant bacteria per granuloma	Total numbers of resistant bacteria per granuloma Total numbers of bacteria per granuloma Fraction of granulomas sterilized Fraction of granulomas that contain only resistant bacteria

#### Resistance selection simulations

Resistance selection simulations are performed in two phases (Fig 3B). First, we generate a repository of 392 granulomas containing only antibiotic-susceptible Mtb by simulating *GranSim* and allowing *in silico* granulomas to form and evolve over 200 days (as described above). At 200 days, we stop the simulations and save the state of the system. To measure the effect of resistant bacteria on treatment efficacy, we randomly select a number of bacteria (5, 20, 100 or all bacteria) within each saved granuloma, and change them to a resistant state. Note that, in line with non-human primate data, the number of bacteria in each granuloma varies, having a mean of 90, and standard deviation of 70 bacteria per granuloma at 200 days post infection. Therefore, in the second phase, we initiate antibiotic treatment in these saved granulomas with resistant Mtb. Treatment with single antibiotics or combination therapy consists of 15 mg/kg dose of INH and 20 mg/kg dose of RIF given daily. These are human-equivalent doses determined in previous non-human primate studies [10, 11, 50]. This strategy provides two advantages: first, it allows the generation of large numbers of granulomas containing specified numbers of resistant bacteria, similar to granulomas observed in the resistance emergence simulations described above, but at a feasible computational cost; and second, it allows the side-by-side comparison of treatment outcomes in identical granulomas with varying numbers of resistant bacteria. We simulate daily treatment with INH and/or RIF to represent the standard 6-month therapy used against active TB [3]. For each granuloma, we track model outcomes that are relevant to resistance selection (Table 1) over 180 days of treatment (from day 200 to 380 post infection).

### Sensitivity and uncertainty analysis

To quantify the influence of resistance acquisition parameters on infection and treatment outcomes we perform sensitivity and uncertainty analyses. We use Latin hypercube sampling to simultaneously sample the multi-dimensional parameter space, and calculate partial rank correlation coefficients (PRCCs) between parameters and model outputs [90]. We sample resistance parameters 40 times and simulate 100 granulomas for each of the parameter sets yielding a total of 4000 simulations. Parameters and ranges are given in Table 2. We calculate PRCCs over time throughout the simulated infections, allowing us to identify important influences that might evolve.

**Table 2:** Drug resistance parameter values and ranges used for sensitivity analysis.

Parameter	Value	Sensitivity analysis ranges	References
Mutation frequency [per base pair per day]	3x10^-6^ (emergence simulations)	3x10^-7^–1x10^-5^	[64]
INH			
Number of resistance-conferring mutations	30	30–300	[91–93]
Relative fitness [fraction of WT growth rate]	0.9	0–1	[25]
Number of compensatory mutations	1	1–10	[25, 26, 82, 94]
RIF			
Number of resistance-conferring mutations	30	30–300	[91, 95]
Relative fitness [fraction of WT growth rate]	0.8	0–1	[73, 74]
Number of compensatory mutations	40	40–400	[22, 80, 94, 95]

### Scaling resistance probabilities from granuloma to host scale

Our simulations are two-dimensional (2D) representations of 3D granulomas in hosts. Furthermore, we simulate individual granulomas while a single host likely has multiple granulomas. A median of 46 granulomas per animal has been measured in non-human primates [49], and humans show involvement in large proportions of their lungs [96].

We therefore scale our results from 2D simulations to obtain host scale estimates. We have previously developed a scaling factor that translates the number of bacteria in a 2D simulation to 3D [10, 57], i.e. if the scaling factor is *S*, then each bacterium in our 2D simulation represents *S* bacteria in 3D. Therefore, for each bacterial division in 2D, we adjust the per-generation mutation probability (*p*_*m*_) to represent *S* divisions in 3D, each associated with their own probability of mutating:

$$p_{m,3D}=1-{\left(1-p_{m}\right)}^{S}$$ (2)

where *p*_*m*_ is defined in Eq 1. This scaling is implemented within *GranSim*, and is therefore being applied to each bacterial division as it occurs in the simulation.

Furthermore, to account for our use of higher mutation frequency (described above), we scale the predicted granuloma resistance frequency (*r*_*G*_) proportionally to the mutational frequency. We assume a linear relationship between mutational frequency and granuloma resistance frequency (Figure A in S1 File). For example, if the predicted *r*_*G*_ in our simulations is 1x10^-1^ for a mutation frequency of 3x10^-6^, then the scaled *r*_*G*_ would be 1x10^-5^ for a mutation frequency of 3x10^-10^.

Finally, we estimate the per host resistance frequency (*r*_*P*_) based on the per granuloma resistance frequency (*r*_*G*_). If a host has *N*_*G*_ granulomas, then the host resistance frequency is:

$$r_{P}=1-{\left(1-r_{G}\right)}^{N_{G}}$$ (3)

### Statistical analysis

Unless otherwise stated the following methods were used for determining statistical significance. To compare number of bacteria per granuloma at specific time points we use one-way ANOVA with Sidak’s multiple comparison test. To compare Kaplan-Meier curves for granuloma sterilization during treatment we use Matel-Cox test, with Benferroni corrections for multiple comparisons. P-values less than 0.05 were considered significant.

## Results

### Resistance emergence dynamics mirror bacterial dynamics

**Fig 4:**
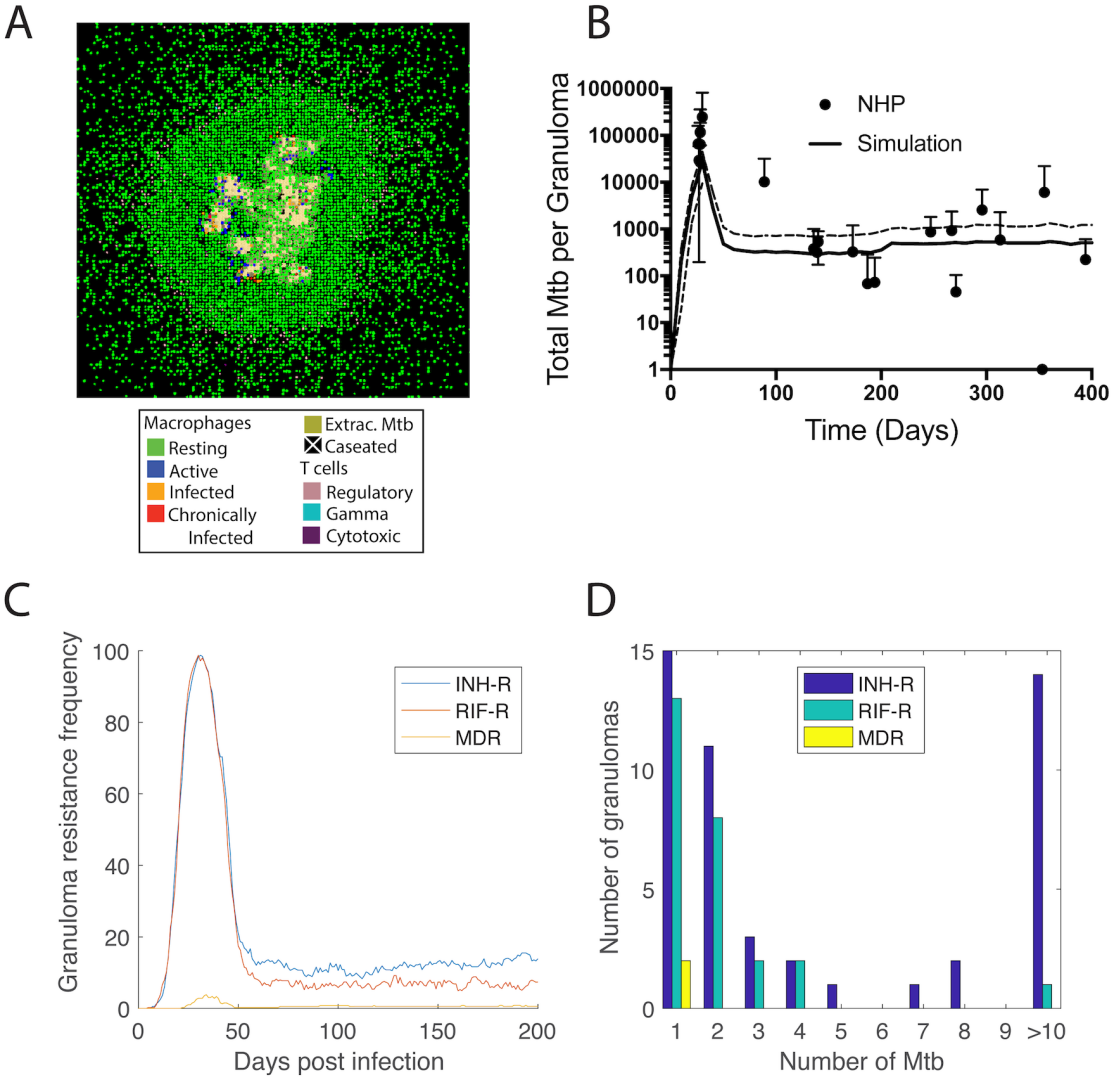
Simulated granuloma dynamics and resistance emergence. The formation of virtual granulomas is an emergent behavior of the model (A). A representative granuloma is shown 200 days post infection (no antibiotic treatment). Colors indicate the location of macrophage populations (resting, active, infected, chronically infected), T cell populations (regulatory, IFN-γ-producing, and cytotoxic), extracellular bacteria and caseum; (B) GranSim is calibrated to bacterial load dynamics observed in Mtb-infected non-human primates [10]. Solid line shows mean and dashed lines show quartiles for 353 simulated granulomas. Data points and error bars show mean and quartiles of between 3 and 84 non-human primates. (C) The granuloma resistance frequency (Box 1) follows bacterial trajectories, peaking at day 30 and leveling off after 60 days. (D) Granulomas that develop resistance have relatively low numbers of resistant bacteria after 200 days of infection, with a few granulomas having higher numbers of INH-R Mtb (up to 165). Resistance emergence is simulated for mutation rates 3x10^-6^ per base pair per day.

Our computational model (Fig 2), tracks host immune cells and bacteria on a grid representing lung tissue, allowing simulation of the formation, evolution and function of lung granulomas. The model also incorporates antibiotics, allowing simulation of plasma PK, distribution of antibiotics from blood vessels into the simulated lung tissue and *in silico* granulomas, and pharmacodynamics (PD) in the form of a concentration dependent killing rate constant (Fig 2A, 2B and 2C). In this work, for the first time we incorporate and are able to track bacterial acquisition of INH and/or RIF resistance and compensatory mutations (Fig 2D) within granulomas. Briefly, following each bacterial division in the model, there is a small probability of resistance conferring mutations occurring. This probability is calculated from experimentally observed mutation rates, number of resistance conferring mutations and growth rates (Methods and Table 2). We assume that when resistant bacteria divide, there is also a probability that the new bacterium acquires a compensatory mutation, resulting in a resistant bacterium that accrues no fitness cost [97]. Emergent behavior in our model is multifold: we observe the formation of *in silico* granulomas (Fig 4A), the appearance of resistant bacteria despite fitness costs, immune killing of bacteria and infected cells, and bacterial dynamics that reflect non-human primate data (Fig 4B).

To predict the dynamics of resistance emergence within granulomas in the absence of antibiotic treatment, we analyze 353 granulomas over 200 days of infection, tracking the number of resistant bacteria per granuloma. At 30 days post infection, we predict that granuloma resistance frequency peaks for INH-R, RIF-R, and MDR Mtb (Fig 4C). This prediction of rapid increase in resistance prior to activation of adaptive immunity is consistent with other studies focused on Mtb evolution during exponential growth [46, 64].

Following the activation of adaptive immunity around 30 days post infection, bacterial loads significantly decrease [7], but no data are available that speak to how this adaptive immune response affects the survival of resistant bacteria that might have emerged during the first 30 days p.i. Our simulations predict that the granulomas resistance frequency (Fig 4C) follows a similar trajectory to the bacterial load (Fig 4B). Granuloma resistance frequencies decrease following activation of adaptive immunity, and then remain relatively stable over the next ~100 days of infection. Our predictions indicate that the granuloma resistance frequencies are 15%, 6% and 1% for INH-R, RIF-R and MDR, respectively. We use these granuloma resistance frequencies to predict host resistance frequencies of 0.7%, 0.3% and 0.05% for INH-R, RIF-R and MDR Mtb, respectively (using Eqs 2 and 3, and assuming each host has on average 46 granulomas [49]). These estimates are in agreement with clinical observations that 0.1% to 15% of pretreatment patients had INH resistant Mtb before widespread use of INH for treatment [84–88], although primary and acquired resistance were not distinguished in those studies.

Of the *in silico* granulomas that contain resistant Mtb, most have relatively low numbers of resistant bacteria after 200 days of infection (Fig 4D), resulting in *hetero-resistant granulomas* (i.e. granulomas with both drug susceptible and drug resistant bacteria). These results suggest that resistant bacteria can exist in the granuloma prior to treatment initiation, even in previously untreated hosts, and they give insight into how the natural infection progression influences resistance dynamics in the absence of treatment [32, 46, 47].

### Mutation rates and fitness costs drive resistance emergence within granulomas

Emergence of resistance is the result of several factors included in our model: mutation frequency, number of resistance conferring mutations, the fitness cost of resistance and number of compensatory mutations. In order to discern the relative contribution of these factors to resistance emergence we perform a sensitivity analysis (see Methods). We sample resistance parameters using Latin hypercube sampling (LHS), and compute partial rank correlation coefficients (PRCC) between model parameters (mutation frequency, number of resistance conferring mutations, the fitness cost of resistance and number of compensatory mutations) and model outputs (granuloma resistance frequency, number of resistant Mtb per granuloma).

**Fig 5:**
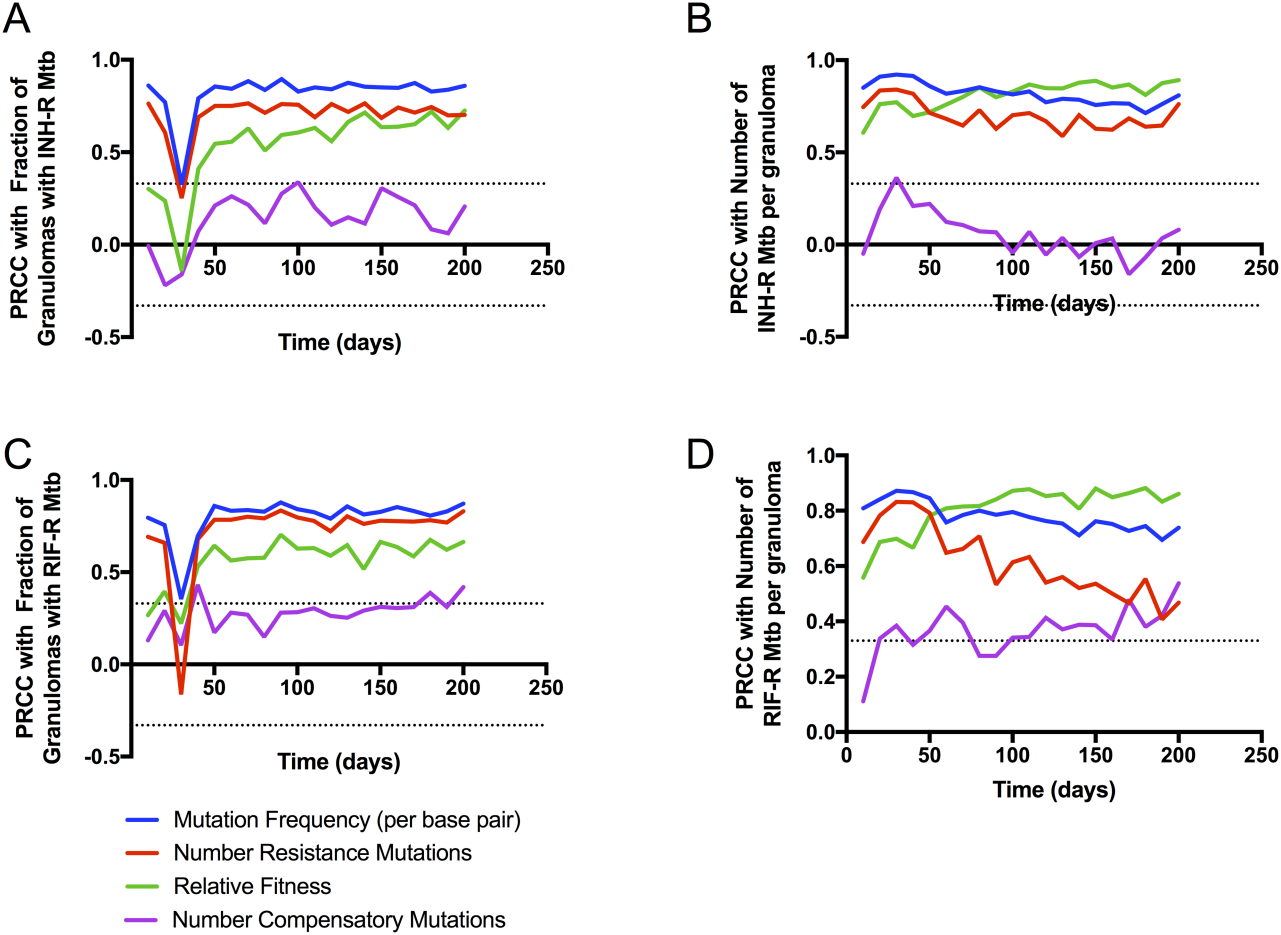
Sensitivity analysis results. PRCCs are shown between model parameters (mutation frequency, number of resistance mutations, relative fitness of resistant Mtb, and number of compensatory mutations) and model outputs of interest (Table 1) (Fraction of Granulomas with INH (A) or RIF (C) resistant Mtb; and the number of INH (B) or RIF (D) resistant Mtb per granuloma). Dotted lines show PRCCs with p-values of 0.05, PRCCs outside these lines are significant.

Sensitivity analysis results indicate that mutation frequency, number of resistance-conferring mutations and fitness costs are the main drivers of number of resistant bacteria per granuloma as well as the granuloma resistance frequency for both INH (Fig 5A and 5B) and RIF (Fig 5C and 5D). Fitness cost has a progressively increasing influence on both these metrics over time, only becoming significantly correlated with granuloma resistance frequency around 50 days post-infection. This temporal dependence indicates that early granuloma resistance frequency is driven by random mutation events, whereas later granuloma resistance frequency is driven by a combination of random mutation events and sustained replication of resistant bacteria. The number of compensatory mutations appear become significantly correlated with the number of RIF resistant Mtb toward the very end of the simulations.

Our results are consistent with other findings that differences between Mtb lineages at the bacterial scale, such as mutation frequency and genetic background determining fitness costs, contribute to differences at the population scale (host resistance frequencies) [32, 34]. In addition, we are able to quantify the time-dependent nature of these contributions.

### Antibiotic-resistant and antibiotic-susceptible Mtb distribute differently within granulomas

It has been shown *in vitro* that the location of Mtb (intracellular vs extracellular) as well as their metabolic state (e.g non-replicating that are located in caseum) can affect their antibiotic susceptibility [98–102]. In addition, antibiotic concentrations are known to vary spatially within granulomas [9–11, 103]. It is therefore of interest to predict the mostly likely location and metabolic state of resistant Mtb for acquired antibiotic-resistant TB. This location will affect the ability of antibiotics to reach resistant bacteria at sufficient concentrations to kill them.

**Fig 6:**
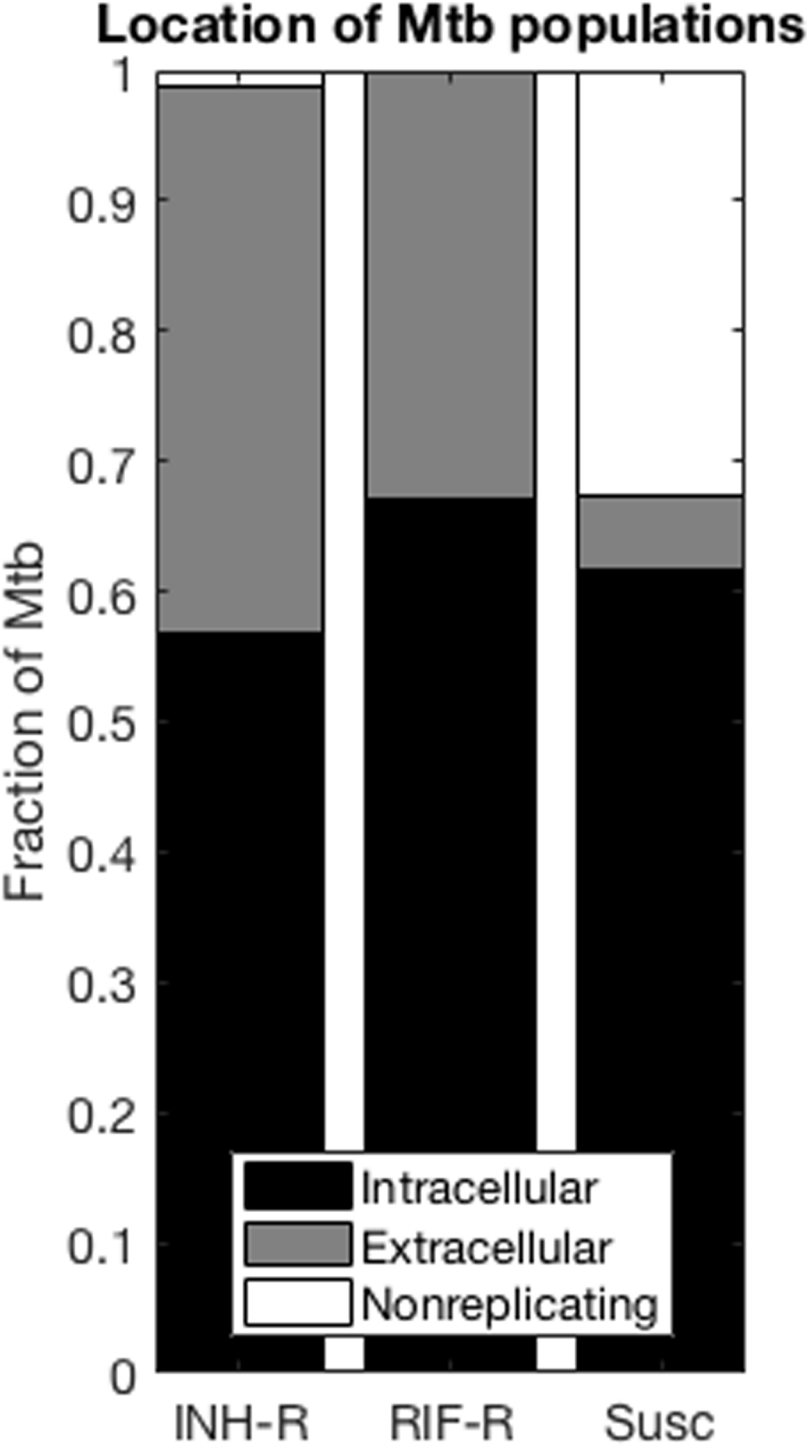
Locations of INH-R, RIF-R and susceptible Mtb within granulomas. Locations include intracellular, extracellular and non-replicating (in caseum) N = 353 granulomas. Locations for MDR Mtb are not shown since too few granulomas contained MDR to enable meaningful analysis.

We examine bacterial phenotypes and location in the collection of 353 simulated granulomas described above. Our results predict that the distribution of resistant Mtb in the three states of intracellular, extracellular, and non-replicating is markedly different from the distribution of susceptible Mtb (Fig 6). Resistant and susceptible Mtb have similar proportions in the intracellular state. However, resistant bacteria are much less likely to be found in the caseum, compared to antibiotic-susceptible Mtb. This is expected, as caseation is driven by infection-induced host cell death, and the lower replication rate constant of resistant Mtb would lead to fewer host cell deaths in areas where resistant bacteria are located. Simulations also predict that the majority of both INH-resistant, RIF-resistant and susceptible Mtb are located intracellularly. These results show how differences between resistant and susceptible bacteria at the bacterial scale contribute to different bacterial distributions at the granuloma scale, effectively enriching certain subpopulations for resistant bacteria. Given the heterogeneous drug exposure of different bacterial subpopulations (Figure B in S1 File) [9, 10], the predominant phenotype and location of naturally emerging resistant bacteria will impact their selection once treatment starts.

### Granuloma sterilization curves shift significantly, even when only a few resistant Mtb are present

The above studies focused on emergence of resistance in the absence of drug treatment. We now turn to simulations of antibiotic treatment to understand how drug regimens (with both single and multiple drugs) and drug concentrations within granulomas affect antibiotic-resistant Mtb. In particular, we ask how these factors affect the selection of pre-existing resistant bacteria. To do this, we introduce varying numbers of resistant bacteria into simulated granulomas immediately prior to treatment (see Fig 3). We predict how the presence of these bacteria prior to treatment affects the response within granulomas under different treatment regimens, compared to the same granulomas lacking any resistant Mtb.

We first predict how INH and RIF affect the selection of resistant bacteria during mono-therapy (treatment with INH or RIF alone). We simulate 392 granulomas using *GranSim* and let them develop for 200 days in the absence of resistance mutations. After 200 days, we randomly select a defined number of bacteria and change them to be resistant, allowing us to study the impact of the initial load of resistant bacteria. Throughout treatment, bacteria still can naturally mutate with a low probability (3x10^-10^ per base pair per day). We vary the number of resistant bacteria present at the start of treatment by increasing them from 5 bacteria up to all bacteria within a simulation (i.e. 5, 10, 20, 100, all). We then initiate daily INH or RIF monotherapy.

**Fig 7:**
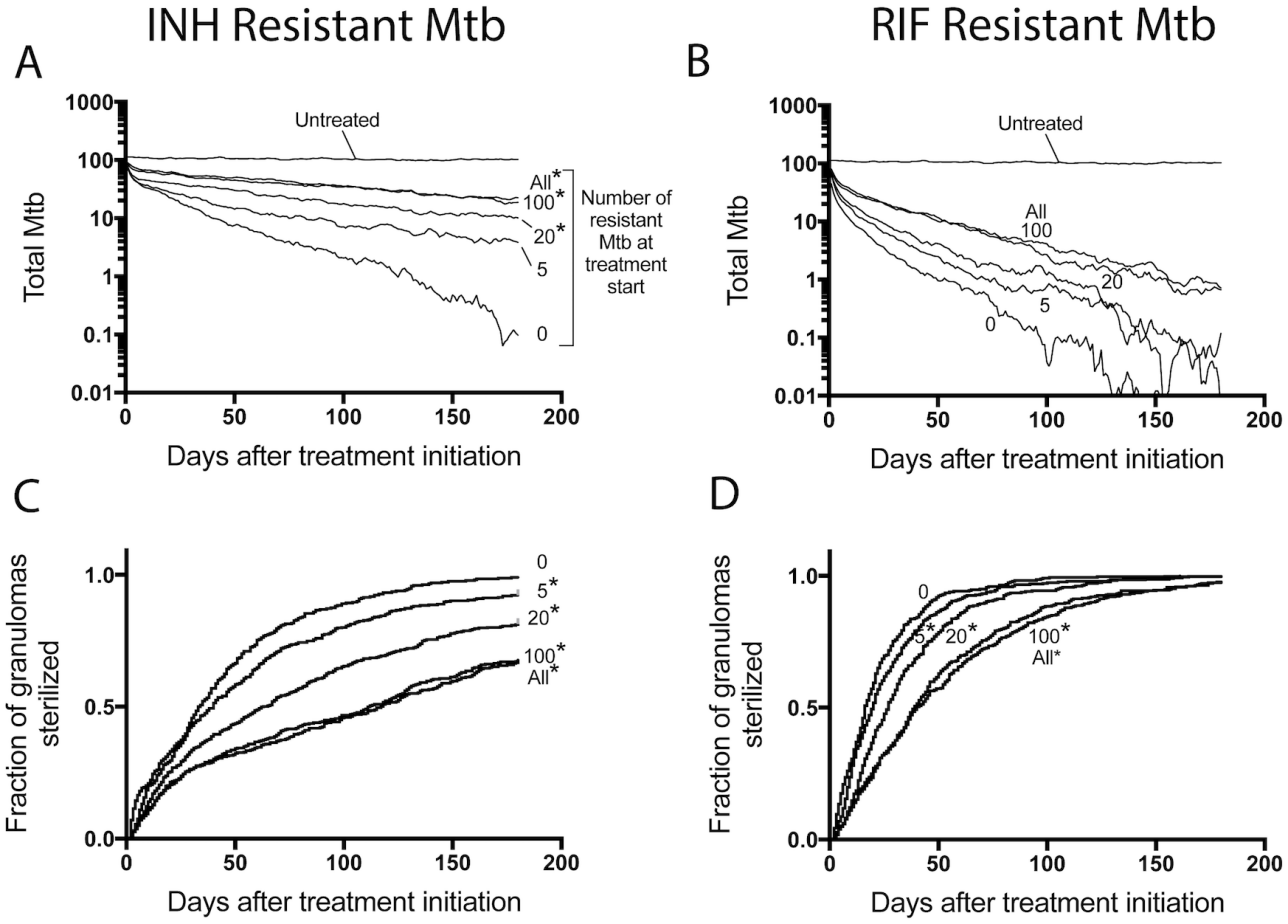
Treatment outcomes under INH- or RIF- monotherapy. In each simulation, a set number of bacteria are selected to be changed to be resistant. Bacteria are selected from each bacterial sub-population (intracellular, extracellular, non-replicating). We include 0, 5, 20, 100 or ALL resistant Mtb into the simulation prior to treatment. Average bacterial load is shown when adding INH-R Mtb and treating with INH monotherapy in (A) or adding RIF-R Mtb and treating with RIF monotherapy in (B). Panels (C) and (D) contain Kaplan-Meier curves for the same simulations in (A) and (B), respectively, showing the fraction of granulomas that sterilized over time. N = 392 granulomas. *: p < 0.05.

Having 20 or more INH-resistant bacteria per granuloma at the start of therapy significantly increases the average bacterial load at the end of 6 months of INH treatment compared to the case with no resistant Mtb (Fig 7A). In contrast, the average bacterial load at the end of RIF monotherapy does not significantly change with increasing numbers of RIF-resistant bacteria present (Fig 7B). As few as 5 INH- or RIF-resistant Mtb significantly shifts the sterilization curves for mono-therapy (Fig 7C and 7D), a result that is even more noteworthy when you consider that hosts have multiple granulomas.

### Pharmacokinetic and pharmacodynamic differences between INH and RIF drive resistance selection

**Fig 8:**
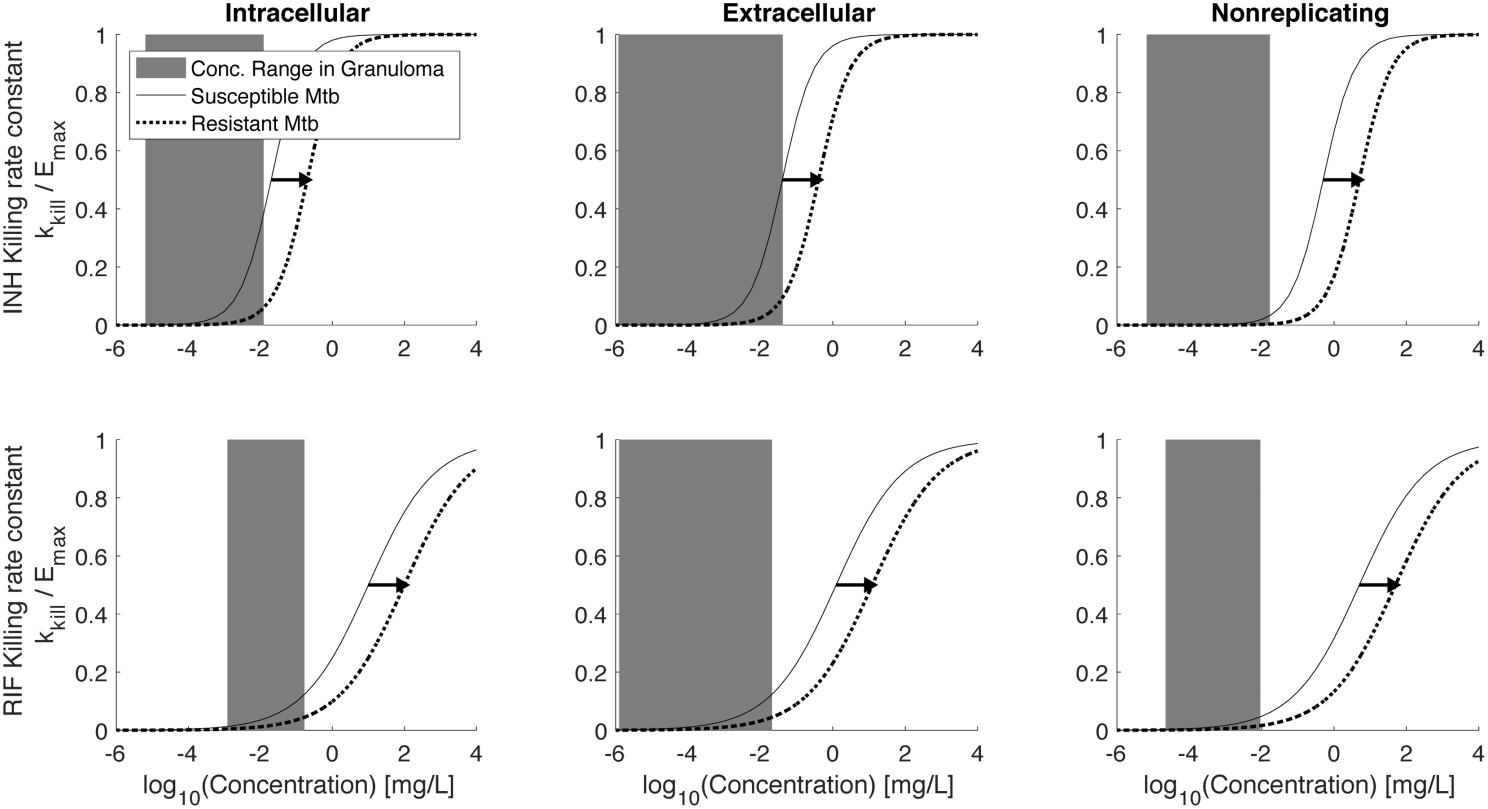
Predicted INH and RIF exposure relative to pharmacodynamic (PD) curves. Solid lines show relative bacterial killing rate constants (k_kill_/E_max_) as a function of concentration for INH susceptible Mtb (A-C) and RIF susceptible Mtb (D-F). Dotted lines show curves for INH- and RIF-resistant Mtb. Gray bars show the range of concentrations that intracellular (A,D), extracellular (B,E) or non-replicating (C,F) Mtb are exposed to over the first 7 days of treatment.

The changes in sterilization curves induced by RIF-R Mtb are smaller than those induced by the same number of INH-R Mtb (compare Fig 7C and 7D). In the model, we account for PK and PD differences between INH and RIF. We can therefore probe how these differences manifest in model outcomes. First consider PD, which we describe in the model as a concentration-dependent killing rate constant (*k*_*kill*_), plotted in Fig 8 as a fraction of the maximum killing rate constant, *E*_*max*_. The two curves shown on each of the graphs represent the PD curve for drug susceptible Mtb (solid curves) and for drug-resistant Mtb (dotted curves); a resistant phenotype induces a shift of the dose response curve to the right [76]. The dose response curves for INH are steeper than for RIF. In addition, the effective concentrations (*C*_*50*_ values) vary with both the antibiotic and the bacterial location and growth state (intracellular, extracellular replicating, or extracellular non-replicating (caseum)). These PD curves are model inputs based on experimental data [10, 12, 100, 101, 104].

The critical model prediction is the concentration of antibiotic to which each bacterial subpopulation is exposed. We are able to predict the average concentration ranges that each bacterial subpopulation is exposed to over 7 days of treatment [10, 11], and relate these ranges to the appropriate PD curves. Concentration ranges are shown as gray shaded areas in Fig 8. Note that at the concentration ranges present within granulomas, shifting the steeper INH curve to the right results in a larger decrease in killing rate constants compared to shifting the less-steep RIF curve. Yet only considering the range of concentrations ignores important PK differences between INH and RIF; i.e. it is also important to know the time spent at various concentrations and where these concentrations fall the PD curves for each bacterial subpopulation. We therefore evaluate INH and RIF concentrations over 7 days of daily dosing within whole granulomas, as well as for each bacterial subpopulation, along with the corresponding killing rate constants (effect curves) (Figure B in S1 File). For each effect curve, we calculate the area under the effect curve (AUC_E_), which is a PD metric of cumulative killing, analogous to the PK metric AUC as a metric of cumulative antibiotic exposure. Ratios of AUC_E_ over 7 days shows that resistance results in 86–90% decrease in INH killing, as compared to 65% decrease in RIF killing (Table 3). Taken together, these results indicate that the observed increased survival of INH resistant Mtb compared to RIF resistant Mtb (Fig 7) are the result of INH having both steeper dose response curves and faster drug elimination (sharp concentration decrease following doses).

**Table 3:** Ratio of AUC_E_ of resistant Mtb vs susceptible Mtb for each bacterial subpopulation for INH or RIF*.

	Intracellular	Extracellular	Non-replicating
INH	0.13	0.14	0.1
RIF	0.35	0.34	0.34

*: A ratio of 0.13 implies that resistant bacteria are only being killed 13% as effectively as susceptible bacteria.

### Resistance selection occurs early during combination therapy

**Fig 9:**
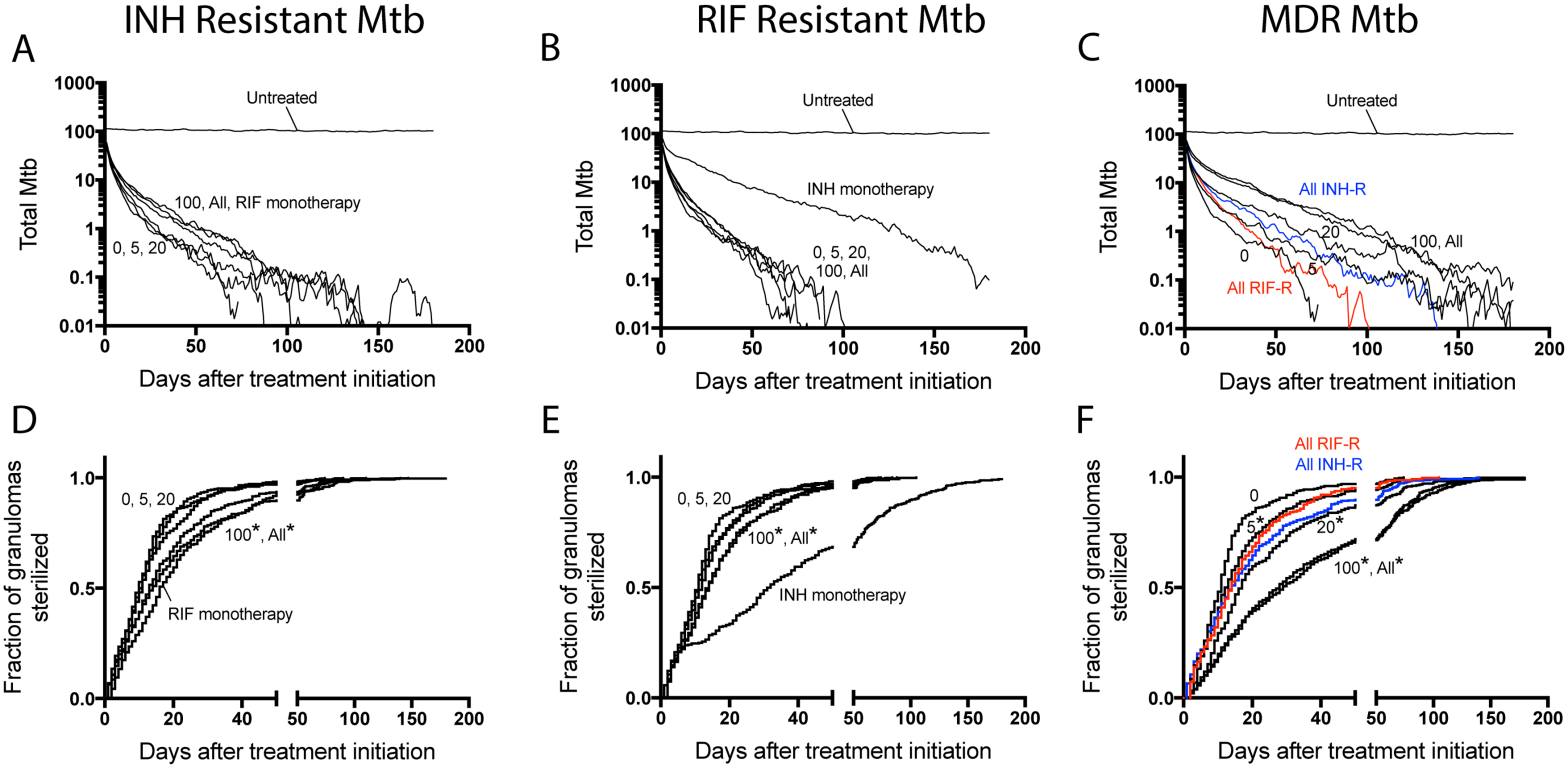
Simulated treatment outcomes under INH and RIF combination therapy. (A-C) The average number of total bacteria per granuloma is shown for INH and RIF combination therapy adding 0, 5, 20, 100 or All INH-R bacteria into granulomas. (A), RIF-R (B) or MDR (C). (D-F) Kaplan-Meier curves for the simulations in (A-C) respectively, showing the fraction of granulomas sterilized over time. N = 392 in silico granulomas.

Since INH and RIF are two of the four antibiotics that make up standard combinations, we next explore how administration of INH and RIF together affects selection of pre-existing resistant Mtb. It is not clear how PK and PD differences between INH and RIF manifest during combination therapy. To directly compare resistance selection of pre-existing resistant Mtb under mono-therapy vs. combination therapy, we start with the same granulomas shown in Fig 7 (i.e. granulomas with varying levels of INH-resistant Mtb or RIF-resistant Mtb introduced at day 200), but now treat each granuloma with a combination of INH and RIF (Fig 9A, 9B, 9D and 9E). We also examine the effect of combination treatment on MDR Mtb (Fig 9C and 9F).

As expected, bacterial loads fall under combination therapy (Fig 9A, 9B and 9C); both drugs and the immune system are now acting to kill bacteria. The rates at which granulomas sterilize in the presence of resistant bacteria are significantly slower than when no resistant bacteria are present (Fig 9D and 9E). Note that for granulomas where all Mtb are INH-resistant, the bacterial load and sterilization curves during combination therapy are nearly identical to the simulations with RIF-monotherapy, when INH is not present (Fig 9A and 9D). This is consistent with results in Fig 7 that indicate INH has a limited contribution to killing INH-resistant Mtb. In contrast, for granulomas where all Mtb are RIF-resistant, the bacterial response and sterilization curves under combination therapy are significantly different from simulations with INH monotherapy, when RIF is not present (Fig 9B and 9E). In contrast to INH-resistant or RIF-resistant Mtb, having as few as 5 MDR Mtb significantly changes bacterial load and sterilization curves during combination therapy (Fig 9C and 9F).

**Fig 10:**
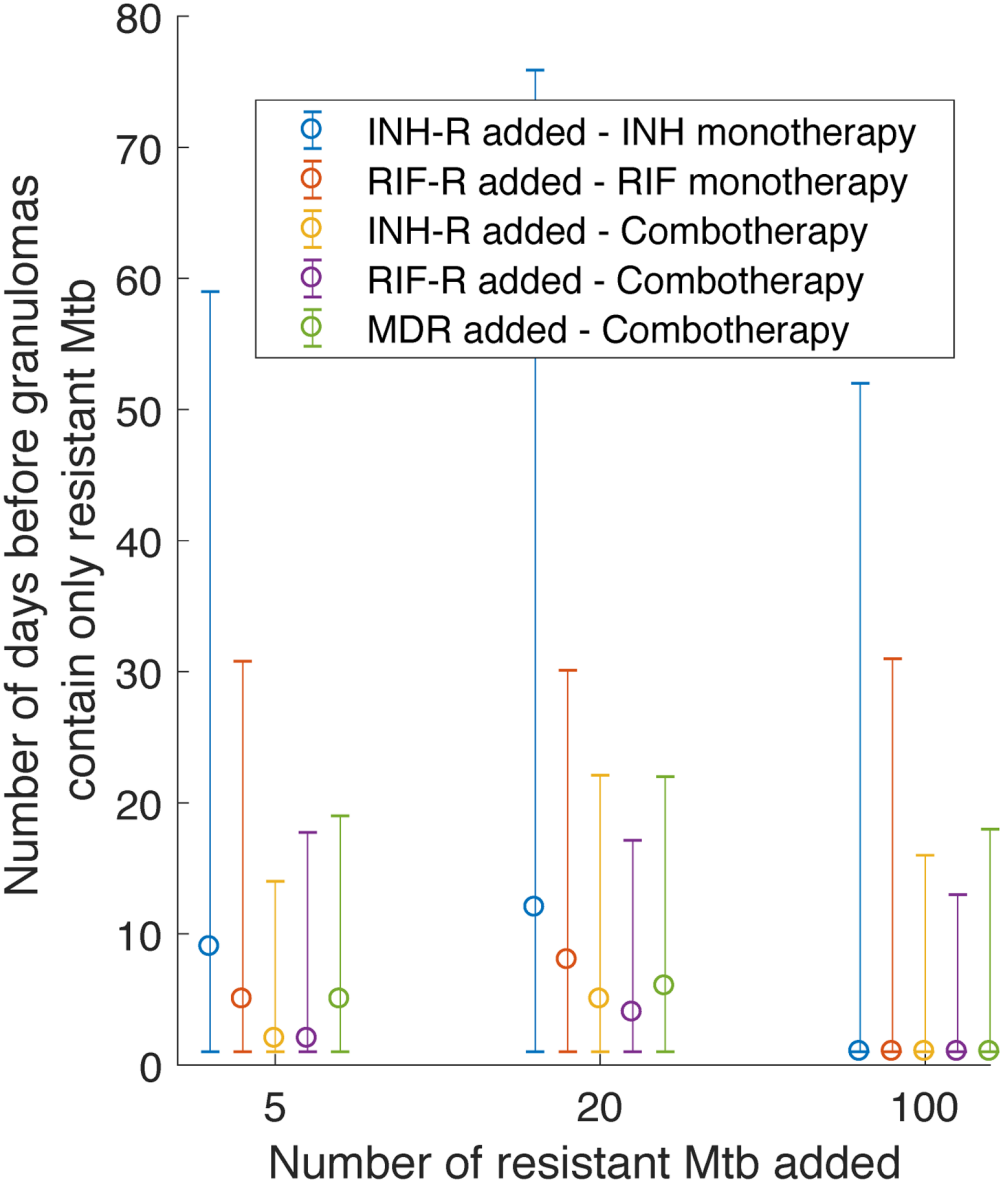
Number of treatment days before granulomas contain only resistant Mtb, when treatment is started with 5, 20 or 100 resistant Mtb present within granulomas. Times are shown for INH-R, RIF-R or MDR bacteria during INH or RIF monotherapy or during combination therapy with INH and RIF. Data points and error bars show median +/- 5th and 95th percentiles for N = 392 in silico granulomas.

Identifying the timing of resistance selection, i.e. how long it takes before the entire surviving bacterial population is antibiotic-resistant, can be a valuable tool to anticipate when patients are at highest risk for developing resistant TB during treatment interruptions. As shown in Fig 10, the median time varies between 1 and 12 days after treatment initiation. In contrast, the median time required for granuloma sterilization varies between 11 and 117 days after treatment initiation (Fig 9). This striking discrepancy in timing illustrates the high risk of antibiotic-resistant TB in patients who interrupt their treatment during the window of time between resistance selection and granuloma sterilization.

## Discussion

Antibiotic-resistant TB is increasing world-wide, even for newly implemented antibiotics [105, 106], adding urgency to the development and responsible implementation of new antibiotics. Our computational model combines host, bacterial and antibiotic interactions over multiple scales to predict resistance emergence and selection in the complex context of lung granulomas. Here, we have demonstrated the approach for INH- and RIF-mono-resistant as well as MDR-Mtb. When expanded to include new antibiotics or new combinations of antibiotics, our approach can help extend their useful life-span by informing the design of resistance-minimizing treatment regimens.

Our approach uniquely integrates mechanisms operating at multiple spatial and temporal scales. Connecting bacterial and granuloma simulation scales, our results predict that the granuloma environment impacts the expansion of resistance: following early expansion the bacterial and granuloma resistance frequencies decrease dramatically, in parallel with total bacterial load, as adaptive immunity develops. This is in contrast to typical resistance predictions that often only consider exponentially growing Mtb [32, 46, 47]. This highlights the importance of host immune mechanisms in controlling natural emergence of resistant, even for multi-drug resistant, Mtb. Our sensitivity analyses indicate that bacterial factors such as Mtb lineage differences in mutation frequency and genetic background [32, 34] significantly affect granuloma resistance frequencies. Our ability to translate these bacterial properties into granuloma-scale resistance predictions, provides an opportunity to make bacterial strain-specific treatment recommendations.

Our simulations integrate PK and PD data to make systems-level predictions for resistance selection for multiple antibiotics. Selection has different dynamics for INH-resistant vs RIF-resistant Mtb within granulomas; these different dynamics are due to a combination of PK and PD differences between INH and RIF. We predict that INH having both steeper dose response curves and faster drug elimination compared to RIF is responsible for INH-resistant Mtb being more likely than RIF-resistant Mtb to survive treatment. These predictions provide a possible mechanism for clinical observations that RIF mono-resistant TB is less frequently found, while INH-mono-resistant TB is more common [6, 107–109]. Our method therefore provides a way to leverage pre-clinical data to quantitatively predict how complex PK and PD interactions allow resistance to develop to different drugs.

Direct experimental validation of our predictions is outside the scope of the current work and would be challenging. The relatively low frequency of resistance per granuloma would require large sample sizes in appropriate animal models which could be prohibitively expensive. While there are several *in vitro* granuloma models being developed [110–112], there is currently no *in vitro* system that accurately represents the long-term dynamics of TB granulomas. Nonetheless, our predictions are indirectly validated by clinical observations that are consistent with previously observed resistance frequencies and differences between INH and RIF [6, 84–88, 107–109].

Connecting granuloma and host simulation scales, we predict that at the granuloma scale (and therefore also at the host scale), hetero-resistance is common. Hetero-resistance is important because it has been linked to poor patient outcomes, and poses significant challenges to diagnosis, especially if the number of resistant Mtb is small, as our simulations suggest [113]. Our host resistance frequency predictions are consistent with observations of INH-R bacteria in untreated patients in the early days of INH implementation [84–88]. As new antibiotics are developed or existing antibiotics are repurposed to treat TB, our simulations could be used to predict the probability of hetero-resistance and how it might contribute resistance emergence.

This work, as well as earlier publications, demonstrate that there is poor exposure of bacteria to INH and RIF within granulomas [9–11]. This observation supports the potential benefits of higher INH doses in treatment of low-level INH-resistant TB (INH-R TB with moderate increases in C_50_ over susceptible TB) [114, 115]. Higher RIF doses have also been proposed and showed promise [116–119], and given the less steep dose response curve of RIF, our results indicate that even modest increases in RIF concentrations in granulomas could affect treatment efficacy. These recommendations need to be weighed against risk of non-compliance due to adverse effects, and treatment costs [120–123]. Our results can therefore inform a larger optimization effort at the population scale by predicting the impact of larger doses on resistance selection given drug dynamics within granulomas.

Connecting host and population simulation scales, we predict that the progression from acquired hetero-resistance in a single host to spread of primary resistant TB in a population likely occurs over time scales longer than a single round of treatment. This is consistent with indications that the XDR-TB outbreak in Tugela Ferry, South Africa likely evolved via multiple rounds of infection and treatment within a community [22]. Furthermore, our prediction of the timing of resistance selection, combined with data showing that treatment interruptions often start around 14 days after treatment initiation [124], provides a framework for public health efforts aimed at keeping patients on their treatment through high-risk times.

Our work also has implications for drug regimen design as new anti-TB antibiotics like delaminid and bedaquiline are integrated into standardized treatment regimens [125, 126]. Using our simulations, risk of resistance can be included in treatment optimization studies [127]. As new regimens are designed, we should assume that some proportion of the population would already harbor a few Mtb resistant to new antibiotics. Furthermore, how these resistant bacteria are selected depends not only on numbers of resistant bacteria, but also on the PK and PD of new drugs, and the existing drugs that are used in combination. Our approach integrates all of these complex interactions into a single computational framework that can make quantitative and drug-specific predictions to minimize resistance that may be translatable for other diseases as well. Important additional complexities that could be considered in future model refinements include bacterial transcriptional changes in response to host immune responses [66], epigenetics [67, 68], inherent bacterial variability [69, 70], lineage differences [32, 71] and efflux pump induction [72].

Finally, our results have some hopeful implications for new anti-TB antibiotics. We predict that prior to wide-spread implementation of a drug, the proportion of patients with pre-existing Mtb resistant to that drug should be low, and in those patients that do harbor resistant bacteria, the numbers of resistant bacteria are likely to be low. It is therefore feasible that host immunity in combination with an optimized combination therapy can sterilize naturally occurring hetero-resistant infections. The global public health infrastructure has improved in the decades since the first implementation of INH and RIF, and it is therefore possible that resistance to new drugs can be limited to individuals, before it spreads as primary resistance.

## Supporting information

S1 File Supplementary figures and tables including: Table A: Host immune and bacterial growth parameters used to generate in silico granulomas; Table B: INH and RIF PK and PD parameters; Figure A: Linear regression between mutation frequency and granuloma resistance frequency; Figure B: Dynamics of antibiotic concentration and bacterial killing rate constants over 7 days of treatment. (PDF)
