# Supplementary material for: Emergence and selection of isoniazid and rifampin resistance in tuberculosis granulomas
Source: PLoS One. 2018 May 10;13(5):e0196322. doi: 10.1371/journal.pone.0196322 (PMC5944939; doi:10.1371/journal.pone.0196322)
Supplement: S1 File — (PDF) [file pone.0196322.s001.pdf]

Supplement to: Emergence and selection of Isoniazid and rifampin resistance in tuberculosis granulomas

GranSim parameters

Table A: Host immune and bacterial growth parameters used to generate in silico granulomas [1,2]

Parameter	Unit*	Value
Bacterial carrying capacity of each grid compartment	Bacteria	115
Intracellular bacterial growth rate	h^{-1}	0.027
Extracellular bacterial growth rate	h^{-1}	0.015
Rate of death of bacteria trapped in caseated compartments	h^{-1}	5.1
Number of host cell deaths causing caseation		9
Time to heal caseation	Days	10
TNF threshold for causing apoptosis	Molecules	1150
Rate of TNF induced apoptosis	s^{-1}	1.7x10-6
Minimum chemokine concentration allowing chemotaxis	Molecules	0.47
Maximum chemokine concentration allowing chemotaxis	Molecules	480
Initial macrophage density	Fraction of grid comp.	0.04
Time steps before a resting macrophage can move	Timesteps	3
Time steps before an activated macrophage can move	Timesteps	19
Time steps before an infected macrophage can move	Timesteps	170
TNF threshold for activating NFkB	Molecules	75
Rate of TNF induced NFkB activation	s^{-1}	1.06x10-5
Number of bacteria resting macrophage can phagocytose	Bacteria	1
Probability of resting macrophage killing bacteria		0.12
Adjustment for killing probability of resting macrophages with NFkB activated		0.2
Number of extracellular bacteria that can activate NFkB	Bacteria	250
Threshold for intracellular bacteria causing chronically infected macrophages	Bacteria	12
Threshold for intracellular bacteria causing macrophage to burst	Bacteria	23
Number of bacteria activated macrophage can phagocytose	Bacteria	5
Probability of an activated macrophage healing a caseated compartment in its Moore neighborhood		0.0055
Probability of a T-cell moving to the same compartment as a macrophage		0.046
IFN γ -producing T-cell probability of inducing Fas/FasL mediated apoptosis		0.035
IFN γ -producing T-cell probability of producing TNF		0.045
IFN γ -producing T-cell probability of producing IFN		0.35
Cytotoxic T-cell probability of killing a macrophage		0.009
Cytotoxic T-cell probability of killing a macrophage and all of its intracellular bacteria		0.7

Cytotoxic T-cell probability of producing TNF		0.05
Regulatory T-cell probability of deactivating activated macrophage		0.008
Time before maximum recruitment rates are reached	Timesteps	980
Macrophage maximal recruitment probability		0.32
Macrophage chemokine recruitment threshold	Molecules	0.86
Macrophage TNF recruitment threshold	Molecules	0.011
Macrophage half sat for TNF recruitment	Molecules	1.6
Macrophage half sat for chemokine recruitment	Molecules	2.2
IFN γ -producing T-cell maximal recruitment probability		0.15
IFN γ -producing T-cell chemokine recruitment threshold	Molecules	0.07
IFN γ -producing T-cell TNF recruitment threshold	Molecules	1.3
IFN γ -producing T-cell half sat for TNF recruitment	Molecules	1.3
IFN γ -producing T-cell half sat for chemokine recruitment	Molecules	2
Cytotoxic T-cell maximal recruitment probability		0.12
Cytotoxic T-cell chemokine recruitment threshold	Molecules	4.5
Cytotoxic T-cell TNF recruitment threshold	Molecules	1.3
Cytotoxic T-cell half sat for TNF recruitment	Molecules	1.2
Cytotoxic T-cell half sat for chemokine recruitment	Molecules	9
Regulatory T-cell maximal recruitment probability		0.03
Regulatory T-cell chemokine recruitment threshold	Molecules	2
Regulatory T-cell TNF recruitment threshold	Molecules	1.7
Regulatory T-cell half sat for TNF recruitment	Molecules	2.2
Regulatory T-cell half sat for chemokine recruitment	Molecules	1.5

Table B: INH and RIF PK and PD parameters

**Parameter Name**	**Units**	**INH**	**RIF**	**Reference**
**Plasma PK parameters**				
Absorption rate constant (k_a)	h^{-1}	3	0.5	Fit to data in [3] guided by values in [4-6]
Intercompartmental clearance rate constant (Q)	L/h/kg	0.1	0.5	Fit to data in [3] guided by values in [4-6]
Plasma volume of distribution (V_p)	L/kg	1	1	Fit to data in [3] guided by values in [4-6]
Peripheral volume of distribution (other organs and tissues) (V_{pe})	L/kg	30	0.5	Fit to data in [3] guided by values in [4-6]
Plasma clearance rate constant (CL)	L/h/kg	1.2	0.4	Fit to data in [3] guided by values in [4-6]
Dose	mg/kg	15	20	[3]
**Lung tissue PK parameters**				
Degradation rate constant, extracellular ($K_{deg,e}$)	s^{-1}	$5.5x10^{-9}$	$7.5x10^{-8}$	Fit to data in [4]
Degradation rate constant, intracellular ($K_{deg,i}$)	s^{-1}	$6.4x10^{-3}$	$6.7x10^{-3}$	Fit to data in [4]

Effective diffusivity (*D*)	cm^2/s	$1.1x10^{-7}$	$7x10^{-7}$	Fit to data in [4] guided by values in [7]
Cellular accumulation ratio (*a*)	-	0.35	18	Fit to data in [4] guided by values in [8-11]
Vascular permeability (*p*)	cm/s	$8.4x10^{-6}$	$1x10^{-5}$	Fit to data in [4] guided by values in [12]
Permeability coefficient (*PC*)	-	0.25	3.3	[4]
**PD parameters**				
C50 for intracellular Mtb ($C_{50,BI}$)	mg/L	0.02	10	[13-16]
C50 for extracellular replicating Mtb ($C_{50,BE}$)	mg/L	0.04	1.23	[13-16]
C50 for extracellular non-replicating Mtb ($C_{50,BN}$)	mg/L	0.5	5	[13-16]
C50 fold increase for resistant Mtb	-	10	10	[17]
Hill constant for intracellular Mtb (H_{BI})	-	1	0.48	[13-16]
Hill constant for extracellular replicating Mtb (H_{BE})	-	1	0.7	[13-16]
Hill constant for extracellular non-replicating Mtb (H_{BN})	-	1	0.7	Assumed same as extracellular replicating
Max activity intracellular ($E_{max,BI}$)	s^{-1}	$7.7x10^{-5}$	$1.1*10^{-4}$	Fit to data in [18] guided by values in [14,15]
Max activity extracellular ($E_{max,BE}$)	s^{-1}	$2.6x10^{-4}$	$5x10^{-4}$	Fit to data in [18] guided by values in [14,15]

Relationship between mutation frequency and granuloma resistance frequency

To quantify the relationship between mutation frequency and granuloma resistance frequency, we vary mutation frequency while holding all other parameters constant as defined in Table 1 in the main text. Results indicate that a linear function could approximate this relationship (Figure S1).

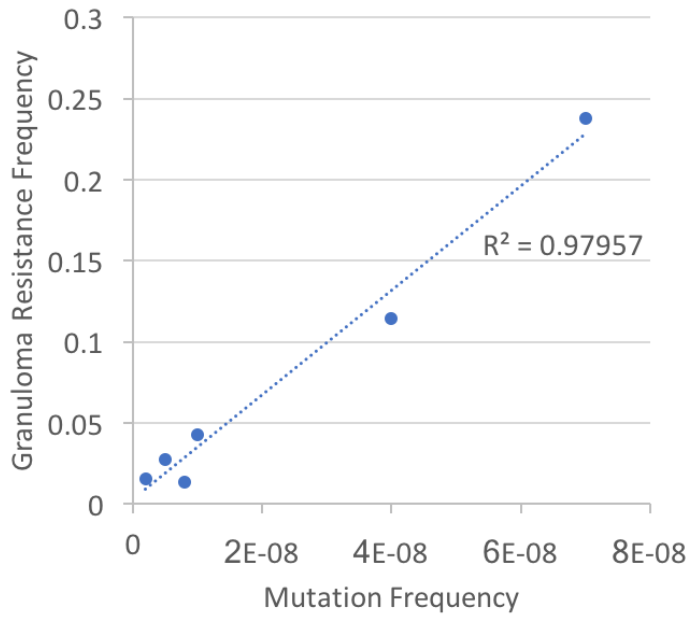

Figure A: Linear regression between mutation frequency and granuloma resistance frequency support the assumption of a linear relationship between these two metrics.

Subpopulation-specific PK-PD within granulomas

To explore the influence of concentration dynamics on bacterial killing within granulomas, we evaluate INH and RIF concentrations over 7 days of daily dosing in the whole granuloma (Figure S2 A,E, left y-axes), or for each bacterial subpopulation (Figure S2 B-D, F-H, left y-axes). Using the dose response curves shown in Figure 6, we calculate the killing rate constants for susceptible Mtb (Figure S2, orange solid lines, right y-axes) and resistant Mtb (orange dotted lines, right y-axes) for each concentration profile. Note the decreasing concentrations that extracellular and non-replicating Mtb are exposed to over the 7 days (Figure S2C,D,G,H), in contrast to the slightly increasing concentrations in the granuloma as a whole (Figure S2A,E). This result indicates that the extracellular and non-replicating Mtb that survive each dose, are the ones that are exposed to lower concentrations.

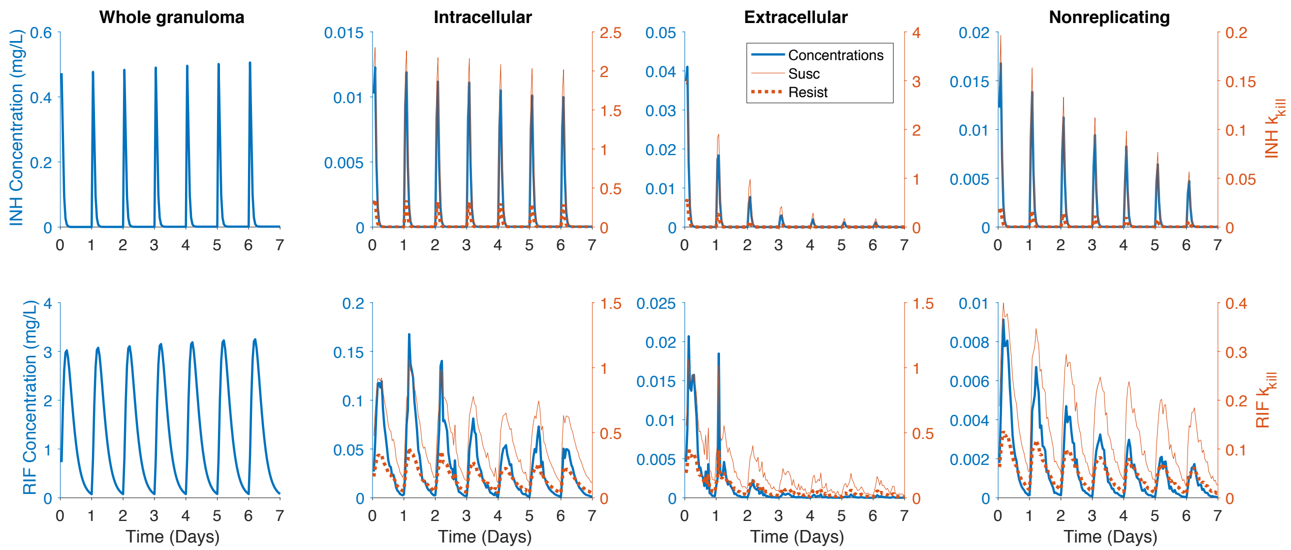

Figure B: Dynamics of antibiotic concentration and bacterial killing rate constants over 7 days of treatment. Blue curves on left y-axes show concentration of INH (A-D) and RIF (E-H) averaged over entire granulomas (A,E) or for intracellular (B,F), extracellular (C,G) or non-replicating (D,H) bacterial subpopulations. Orange curves on right y-axes show bacterial killing rate constants corresponding to the blue curves in each figure for antibiotic susceptible Mtb (solid lines) and resistant Mtb (dotted lines).

References:

1 Cilfone, N. A. *et al.* Computational modeling predicts interleukin-10 control of lesion sterilization by balancing early host-immunity-mediated antimicrobial responses with caseation during mycobacterium tuberculosis infection. *J Immunol* **194**, 664-677, doi:10.4049/jimmunol.1400734 (2015).

2 Pienaar, E. *et al.* A computational tool integrating host immunity with antibiotic dynamics to study tuberculosis treatment. *J Theor Biol* **367**, 166-179, doi:10.1016/j.jtbi.2014.11.021 (2015).

3 Lin, P. L. *et al.* Metronidazole prevents reactivation of latent Mycobacterium tuberculosis infection in macaques. *Proceedings of the National Academy of Sciences of the United States of America* **109**, 14188-14193, doi:10.1073/pnas.1121497109 (2012).

4 Kjellsson, M. C. *et al.* Pharmacokinetic evaluation of the penetration of antituberculosis agents in rabbit pulmonary lesions. *Antimicrobial agents and chemotherapy* **56**, 446-457, doi:10.1128/AAC.05208-11 (2012).

5 Wilkins, J. J. *et al.* Variability in the population pharmacokinetics of isoniazid in South African tuberculosis patients. *British journal of clinical pharmacology* **72**, 51-62, doi:10.1111/j.1365-2125.2011.03940.x (2011).

6 Wilkins, J. J. *et al.* Population pharmacokinetics of rifampin in pulmonary tuberculosis patients, including a semimechanistic model to describe variable absorption. *Antimicrobial agents and chemotherapy* **52**, 2138-2148, doi:10.1128/AAC.00461-07 (2008).
7 Pruijn, F. B., Patel, K., Hay, M. P., Wilson, W. R. & Hicks, K. O. Prediction of Tumour Tissue Diffusion Coefficients of Hypoxia-Activated Prodrugs from Physicochemical Parameters. *Australian Journal of Chemistry* **61**, 687-693 (2008).
8 Jeena, P. M., Bishai, W. R., Pasipanodya, J. G. & Gumbo, T. In silico children and the glass mouse model: clinical trial simulations to identify and individualize optimal isoniazid doses in children with tuberculosis. *Antimicrobial agents and chemotherapy* **55**, 539-545, doi:10.1128/AAC.00763-10 (2011).
9 Mor, N., Simon, B., Mezo, N. & Heifets, L. Comparison of activities of rifapentine and rifampin against Mycobacterium tuberculosis residing in human macrophages. *Antimicrobial agents and chemotherapy* **39**, 2073-2077 (1995).
10 Forsgren, A. & Bellahsene, A. Antibiotic accumulation in human polymorphonuclear leucocytes and lymphocytes. *Scandinavian journal of infectious diseases. Supplementum* **44**, 16-23 (1985).
11 Ziglam, H. M., Baldwin, D. R., Daniels, I., Andrew, J. M. & Finch, R. G. Rifampicin concentrations in bronchial mucosa, epithelial lining fluid, alveolar macrophages and serum following a single 600 mg oral dose in patients undergoing fibre-optic bronchoscopy. *The Journal of antimicrobial chemotherapy* **50**, 1011-1015 (2002).
12 Schmidt, M. M. & Wittrup, K. D. A modeling analysis of the effects of molecular size and binding affinity on tumor targeting. *Molecular cancer therapeutics* **8**, 2861-2871, doi:10.1158/1535-7163.MCT-09-0195 (2009).
13 de Steenwinkel, J. E. *et al.* Time-kill kinetics of anti-tuberculosis drugs, and emergence of resistance, in relation to metabolic activity of Mycobacterium tuberculosis. *The Journal of antimicrobial chemotherapy* **65**, 2582-2589, doi:10.1093/jac/dkq374 (2010).
14 Jayaram, R. *et al.* Pharmacokinetics-pharmacodynamics of rifampin in an aerosol infection model of tuberculosis. *Antimicrobial agents and chemotherapy* **47**, 2118-2124 (2003).
15 Jayaram, R. *et al.* Isoniazid pharmacokinetics-pharmacodynamics in an aerosol infection model of tuberculosis. *Antimicrobial agents and chemotherapy* **48**, 2951-2957, doi:10.1128/AAC.48.8.2951-2957.2004 (2004).
16 Gumbo, T. *et al.* Isoniazid bactericidal activity and resistance emergence: integrating pharmacodynamics and pharmacogenomics to predict efficacy in different ethnic populations. *Antimicrobial agents and chemotherapy* **51**, 2329-2336, doi:10.1128/AAC.00185-07 (2007).
17 Schon, T. *et al.* Evaluation of wild-type MIC distributions as a tool for determination of clinical breakpoints for Mycobacterium tuberculosis. *The Journal of antimicrobial chemotherapy* **64**, 786-793, doi:10.1093/jac/dkp262 (2009).
18 Lin, P. L. *et al.* Radiologic responses in cynomolgous macaques for assessing tuberculosis chemotherapy regimens. *Antimicrobial agents and chemotherapy*, doi:10.1128/AAC.00277-13 (2013).
